# Supplementary material for: Crystal structure of human RIOK2 bound to a specific inhibitor
Source: Open Biol. 2019 Apr 17;9(4):190037. doi: 10.1098/rsob.190037 (PMC6501643; doi:10.1098/rsob.190037)
Supplement: Supplementary Figures and methods for MD simulations [file rsob190037supp1.docx]

Crystal structure of human RIOK2 bound to a specific inhibitor

Supplementary Material

Contents

[MD simulations of RIOK2 dimer or monomer interactions with compound 9 1](#_Toc527832082)

[Supplementary Figure S1 – MD simulation of RIOK2 dimer interaction with compound 9 2](#_Toc527832083)

[Supplementary Figure S2 – MD simulation of RIOK2 monomer interaction with compound 9 3](#_Toc527832084)

## MD simulations of RIOK2 dimer or monomer interactions with compound 9

System preparation and setup for OPLS3 force field were exactly as described in (1) with Schrodinger Maestro version 17.3. In the dimer the dynamics reveals oscillation around a single torsion C7-C8 (see Figure S1) for the naphthyl group around 90 (as seen in the X-ray structure), while having distribution around -90 (X-ray) and -180/180 for the C6-C7 torsion. Stacking with His201 is maintained over 84% of the time. In the monomer the dynamics shows oscillation around 90 (X-ray) for C7-C8 torsion but also around -90 (180 degree naphthyl flip) and distribution around -90 (X-ray) for C6-C7 torsion. Interactions of compound **9** with Ile191, Ala121 and Met101 are maintained during the dynamics for both monomer and dimer.

1. Gao, C., Desaphy, J., and Vieth, M. (2017) Are induced fit protein conformational changes caused by ligand-binding predictable? A molecular dynamics investigation. *J. Comput. Chem.* **38**, 1229–1237

## Supplementary Figure S1 – MD simulation of RIOK2 dimer interaction with compound 9


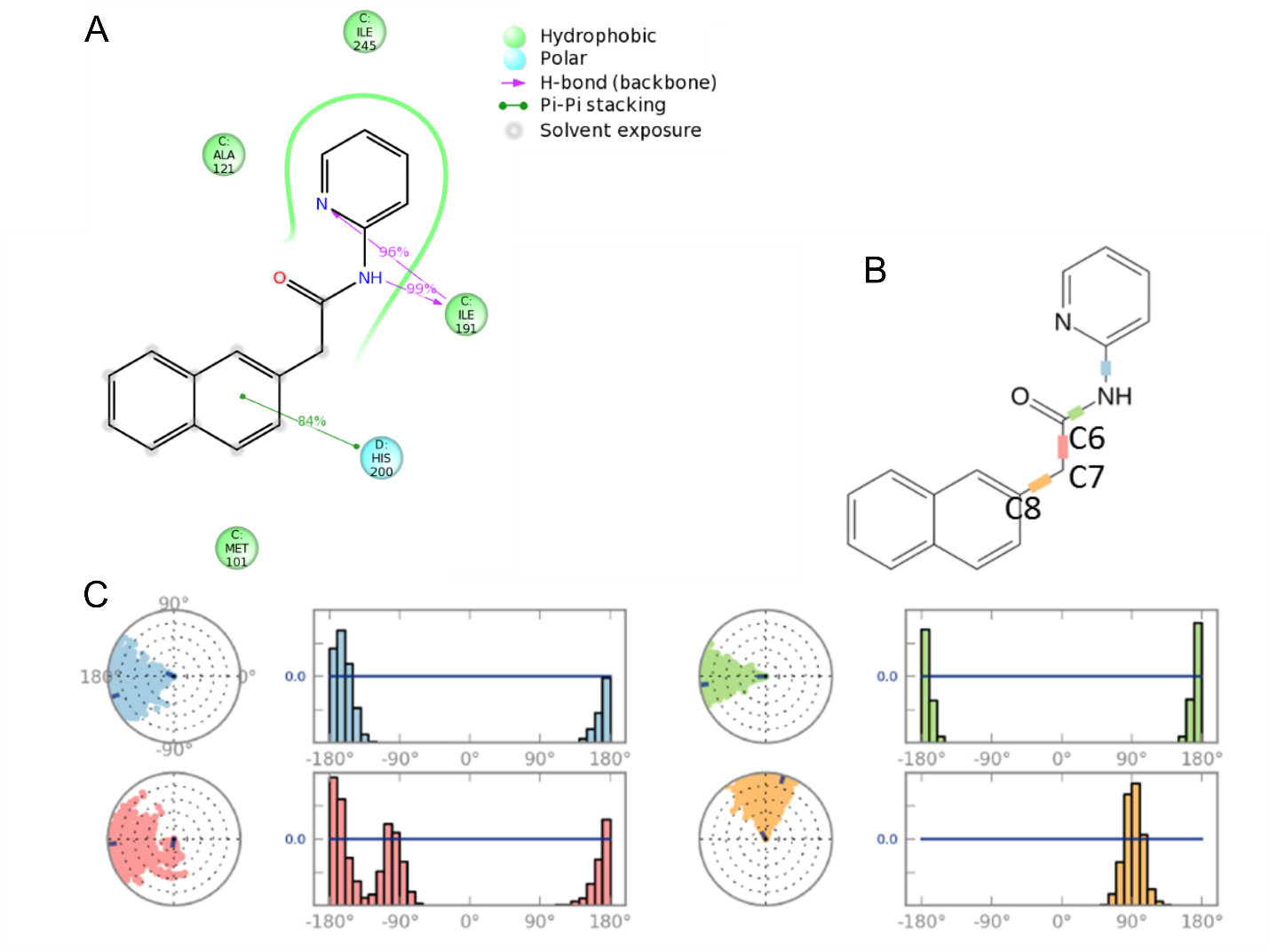


**Figure S1.** 200ns Molecular Dynamics of RIOK2 dimer focusing on compound **9** interactions and torsional space. **(A)** Simulation interaction diagram for compound **9** in dimer. **(B)** The indicated color-coded rotatable bonds for the ligand were analysed. **(C)** Dial (or radial) plots describe the conformation of the torsion throughout the course of the simulation. The beginning of the simulation is in the center of the radial plot and the time evolution is plotted radially outwards. The bar plots summarize the data on the dial plots, by showing the probability density of the torsion. If torsional potential information is available, the plot also shows the potential of the rotatable bond (by summing the potential of the related torsions). The values of the potential are on the left Y-axis of the chart, and are expressed in kcal/mol. Looking at the histogram and torsion potential relationships may give insights into the conformational strain the ligand undergoes to maintain a protein-bound conformation.

## Supplementary Figure S2 – MD simulation of RIOK2 monomer interaction with compound 9


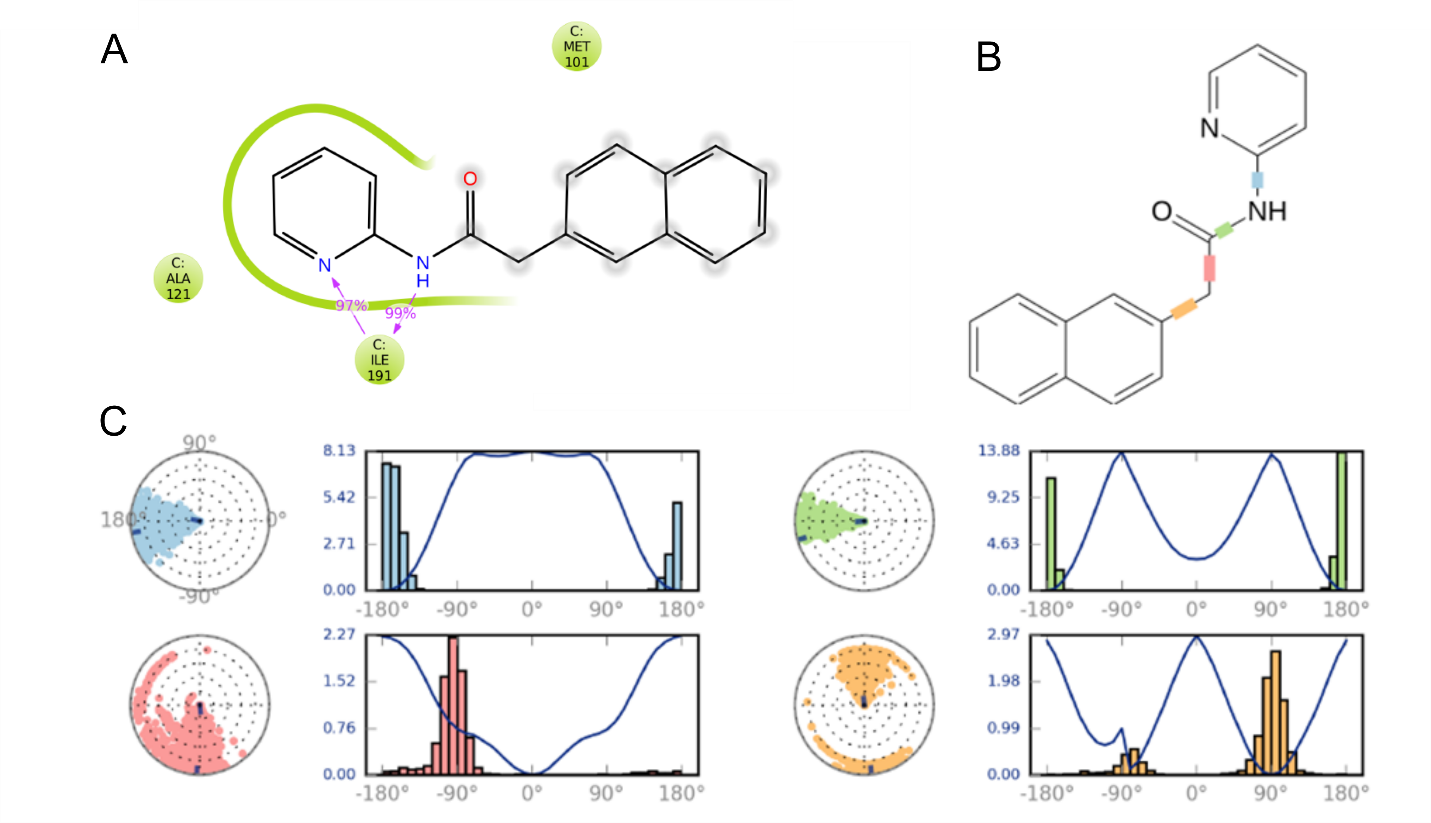


**Figure S2.** 200ns Molecular Dynamics of RIOK2 monomer focusing on compound **9** interactions and torsional space. **(A)** Simulation interaction diagram for compound **9** in dimer. **(B)** The indicated color-coded rotatable bonds for the ligand were analysed. **(C)** Dial (or radial) plots describe the conformation of the torsion throughout the course of the simulation, for comparison with the equivalent simulation data for a RIOK2 dimer in **Supplementary Figure 1C.**
